# Effects of dietary inclusion of *Hermetia illucens* and *Pomacea canaliculata* with *Bacillus amyloliquefaciens* supplementation on performance and egg quality of laying Mojosari ducks

**DOI:** 10.14202/vetworld.2026.2568-2584

**Published:** 2026-06-25

**Authors:** Wizna Wizna, Yan Heryandi, Romi Andika, Fajri Maulana, Satri Yusasra Agasi, Wilghi Deva Ridilan, Shinthia Rahmi, Mutia Nabila

**Affiliations:** 1 Department of Animal Feed and Technology, Faculty of Animal Science, Universitas Andalas, Padang, West Sumatra, Indonesia; 2 Department of Technology and Livestock Production, Faculty of Animal Science, Universitas Andalas, Padang, West Sumatra, Indonesia; 3 Doctoral Program in Animal Science, Faculty of Animal Science, Universitas Andalas, Padang, West Sumatra, Indonesia; 4 Department of Agricultural Industrial Technology, Study Program of Animal Feed Technology, Politeknik Negeri Tanah Laut, South Kalimantan, Indonesia; 5 Student Faculty of Animal Science, Universitas Andalas, Padang, West Sumatra, Indonesia

**Keywords:** alternative protein source, *Bacillus amyloliquefaciens*, egg quality, egg production, *Hermetia illucens*, laying ducks, Mojosari ducks, *Pomacea canaliculate*

## Abstract

**Background and Aim:**

The rising costs and limited availability of conventional feed ingredients, particularly fishmeal and soybean meal, have prompted the exploration of alternative protein sources for sustainable laying duck production. Hermetia illucens larvae and Pomacea canaliculata (Lamarck) are locally available, protein-rich resources with considerable nutritional potential; however, their use in laying duck diets remains limited due to concerns about nutrient digestibility and feed efficiency. Supplementation with probiotics such as Bacillus amyloliquefaciens may improve nutrient utilization and support laying performance when unconventional feed ingredients are used. Therefore, this study evaluated the effects of dietary inclusion of an H. illucens larvae and P. canaliculata (HI–PCL) meal blend supplemented with B. amyloliquefaciens on the performance, egg quality, and economic efficiency of laying Mojosari ducks.

**Materials and Methods:**

A total of 200 laying Mojosari ducks aged 25 weeks were used in a 6-week feeding trial arranged in a completely randomized design with four dietary treatments and five replicates per treatment. The treatments consisted of different inclusion levels of an HI–PCL meal blend (80% HI and 20% PCL) in the diets: 0%, 5%, 10%, and 15%. All ducks received *B. amyloliquefaciens* supplementation through drinking water throughout the experiment. Parameters evaluated included feed intake, daily egg production, egg weight, egg mass, feed conversion ratio (FCR), yolk index, Haugh unit, yolk color, egg dimensions, and income over feed cost (IOFC). Data were analyzed using analysis of variance followed by Duncan’s Multiple Range Test at p < 0.05.

**Results:**

Dietary inclusion of the HI–PCL meal blend significantly affected (p < 0.05) feed intake, egg production, egg weight, egg mass, FCR, yolk index, Haugh unit, yolk color, and IOFC. Ducks fed the 15% HI–PCL diet showed the best productive performance, with daily egg production of 72.38%, egg weight of 68.96 g, egg mass of 49.66 g/bird/day, and FCR of 3.14. Yolk color intensity also increased significantly at the 15% inclusion level. However, egg length and egg width were not significantly affected (p > 0.05) by dietary treatment. The highest IOFC value (Indonesian rupiah [IDR] 847,176.64) was recorded in the 15% HI–PCL treatment.

**Conclusion:**

Inclusion of a 15% HI–PCL meal blend supplemented with *B. amyloliquefaciens* improved laying performance, feed efficiency, yolk pigmentation, and economic return in Mojosari ducks without adversely affecting egg physical characteristics. Under the formulation used in this study, the HI–PCL blend completely replaced fish meal and reduced soybean meal inclusion by approximately 40%, demonstrating its potential as a sustainable alternative protein source for laying duck diets.

## INTRODUCTION

Demand for poultry products, particularly eggs, has increased globally in line with population growth and the need for affordable sources of animal protein. The poultry industry continues to expand to meet rising nutritional needs worldwide, especially in developing countries facing food security challenges [1]. In many developing countries, laying ducks are an important component of the livestock sector because of their adaptability to diverse production systems and their contribution to local food security through egg supply [2]. Similarly, global demand for poultry eggs has continued to increase with population growth and rising consumption of animal protein, particularly in developing regions [3].

Feed is the largest cost component in duck production, often accounting for more than 60%–70% of total production costs [4]. Reliance on conventional protein sources such as soybean meal and fish meal presents major challenges, including global price fluctuations, limited supply, and environmental sustainability concerns [5]. These constraints emphasize the need to explore alternative feed ingredients derived from locally available resources with competitive nutritional value [6].

*Hermetia illucens* has been widely reported as a promising alternative protein source for poultry diets, particularly as a substitute for fish meal or soybean meal [7]. It contains relatively high crude protein (37%–45%) and a high fat fraction that can contribute to dietary energy [8]. The essential amino acid profile of *H. illucens* has been reported to be comparable to that of conventional protein ingredients, suggesting its potential to support poultry amino acid requirements when properly formulated [9]. In addition, *H. illucens* larvae can rapidly bioconvert organic waste into nutritionally valuable biomass [10]. Thus, the use of *H. illucens* not only provides an alternative protein source but also yields by-products that can be used as organic fertilizer, thereby supporting sustainable agriculture [11].

However, the inclusion of *H. illucens* in poultry diets is associated with several limitations. A major constraint is the presence of chitin in the larval exoskeleton, which is poorly digested and may bind nutrients, potentially reducing protein digestibility and overall nutrient utilization efficiency in poultry [12]. Studies evaluating the protein quality of *H. illucens* have demonstrated that reduction of the chitin fraction can improve protein digestibility [13]. Variability in fat content and fiber fractions may also affect palatability and digestibility, which may explain why poultry performance in response to *H. illucens* often depends on inclusion level [7]. In laying poultry, different inclusion levels of *H. illucens* have been reported to produce non-linear responses in performance and egg quality parameters [14].

*Pomacea canaliculata* (Lamarck), widely recognized as a pest that damages rice seedlings (*Oryza sativa* L.) in many Asian and tropical regions, requires sustainable control strategies [15]. Nevertheless, *P. canaliculata* has considerable potential as a local animal protein source because its biomass is abundant in paddy fields [16]. It contains relatively high protein, substantial mineral content, and essential amino acids that may support livestock nutrient requirements [17]. The inclusion of *P. canaliculata* meal in duck diets can be applied at certain levels without compromising performance [18]. Moreover, the use of *P. canaliculata* as a feed ingredient offers ecological value by helping to reduce pest populations and supporting integrated crop–livestock systems [16].

Combining *H. illucens* and *P. canaliculata* in poultry diets may provide complementary benefits, as both are alternative protein sources with distinct nutrient profiles and bioactive components. *H. illucens* is known for its high protein content and relatively favorable essential amino acid profile [19]. However, studies specifically evaluating the combined use of *H. illucens* and *P. canaliculata* remain limited. Most studies involving *H. illucens* in poultry nutrition have focused on single-ingredient substitution for soybean meal or fish meal [20].

Probiotics are defined as live microorganisms that, when administered in adequate amounts, confer health benefits to the host, and their use as functional feed additives in poultry nutrition has increased considerably [21]. Probiotics generally act by modulating the intestinal microbiota, enhancing intestinal barrier integrity, stimulating immune responses, and improving antioxidant status, ultimately contributing to more efficient nutrient utilization [22]. One widely studied probiotic is *Bacillus amyloliquefaciens*, a spore-forming bacterium that is relatively stable during storage and capable of surviving gastrointestinal conditions in poultry [23]. Supplementation with *B. amyloliquefaciens* in laying hens has been reported to improve production performance, enhance egg quality, and support physiological status and gut health by modulating microbiota composition and improving intestinal barrier function [24].

Although the individual benefits of *H. illucens*, *P. canaliculata*, and *B. amyloliquefaciens* have been extensively reported, scientific evidence integrating these three components into a single feeding strategy for laying ducks remains scarce. Previous studies have primarily focused on the partial replacement of soybean meal or fish meal using *H. illucens* alone, whereas limited information is available regarding the synergistic utilization of *H. illucens* and *P. canaliculata* as complementary local protein sources. Furthermore, the effects of combining these alternative feed ingredients with probiotic supplementation on laying performance, egg quality, nutrient utilization, and economic efficiency in Mojosari ducks have not been comprehensively investigated. In addition, the optimal inclusion level of the *H. illucens–P. canaliculata* (HI–PCL) meal blend under probiotic-supplemented feeding systems remains unclear. Therefore, further investigation is required to evaluate whether the combined application of the HI–PCL meal blend and *B. amyloliquefaciens* can provide sustainable nutritional and productive benefits while reducing dependence on conventional protein ingredients in laying duck diets.

Therefore, this study aimed to evaluate the effects of dietary inclusion of an HI–PCL meal blend supplemented with *B. amyloliquefaciens* on the performance, egg quality, and economic efficiency of laying Mojosari ducks. The study also aimed to determine the optimal inclusion level of the HI–PCL meal blend as a sustainable alternative protein source that can partially or completely replace conventional protein ingredients, such as fish meal and soybean meal, in laying duck diets. The findings of this study are expected to provide scientific evidence supporting the development of efficient, sustainable, and locally resource-based feeding strategies for laying duck production systems.

## MATERIALS AND METHODS

### Ethical approval

Ethical approval for this study was obtained from the Ethics Committee of Universitas Andalas, Padang, West Sumatra, Indonesia, under approval number 25/UN16.10.D.KEPK-FF/2026. All experimental procedures involving animals were conducted in accordance with institutional guidelines for animal care and use, as well as Indonesian regulations concerning animal welfare and ethical standards in livestock research. The study involved only routine feeding management and non-invasive husbandry practices, without any surgical manipulation or procedures that would cause unnecessary stress or pain to the animals. Throughout the experimental period, all efforts were made to maintain animal welfare, including proper housing, environmental management, health monitoring, and provision of adequate feed and drinking water. The ethical approval reference number will be provided upon official issuance by the institution.

### Study period and location

The study was conducted for 6 weeks at the experimental poultry facility of the Faculty of Animal Science, Universitas Andalas, Padang, West Sumatra, Indonesia. Laboratory analyses for nutrient composition and feed evaluation were conducted at the Laboratory of Animal Nutrition and Feed Technology, Faculty of Animal Science, Universitas Andalas, Padang, Indonesia. The experiment was carried out under tropical environmental conditions, with average daily temperatures of 27-30°C and relative humidity of 70-85%.

### Study design

This study employed an experimental approach with a completely randomized design, comprising four dietary treatments and five replicates per treatment. Each replicate consisted of 10 laying Mojosari ducks housed in a single pen, for a total of 200 ducks. The treatments were based on different inclusion levels of the HI–PCL meal blend in the diets as follows:

A: 0% HI–PCL meal blend
B: 5% HI–PCL meal blend
C: 10% HI–PCL meal blend
D: 15% HI–PCL meal blend

All experimental diets were formulated to be iso-nitrogenous and iso-caloric, containing approximately 17% crude protein and 2,700 kcal/kg metabolizable energy. All ducks received *B. amyloliquefaciens* supplementation in the drinking water throughout the experimental period, according to the supplementation protocol described below.

### Materials

This study used 200 female Mojosari laying ducks aged 25 weeks, which were reared for 6 weeks in floor-litter pens measuring 200 × 100 × 60 cm per pen. The Mojosari ducks were obtained from the Superior Livestock Breeding Center, Pelaihari, South Kalimantan, Indonesia. At the start of the experiment, the initial body weight was approximately 1.65 ± 0.12 kg/bird (mean ± SD), whereas after 6 weeks of rearing, the final body weight reached approximately 1.72 ± 0.13 kg/bird.

The number of animals used was determined using a completely randomized design with five replicates per treatment, a common approach in poultry nutrition studies to ensure adequate statistical power to detect dietary treatment effects. In addition, the use of 8–12 birds per experimental unit is frequently adopted in laying performance trials to reduce individual variation within each unit.

The experimental facility consisted of 20 pens with 10 ducks per pen. The floor area of each pen was 2 m², resulting in a stocking density of 5 birds/m². Rice husk was used as litter bedding with an initial depth of approximately 8–10 cm. The litter was stirred daily and partially replaced when wet to maintain dry and hygienic conditions throughout the experimental period. Each pen was equipped with feeders, drinkers, a brooder, and a digital scale for measuring feed intake and egg weight.

At the beginning of the rearing period, all ducks were clinically healthy and had vaccination histories against Newcastle disease and infectious bronchitis, in accordance with the source facility’s vaccination program. A 16 h light/8 h dark (16L:8D) lighting program was used throughout the study. White light-emitting diode lamps (approximately 10–12 W) were installed in each pen, and light intensity at the birds’ head level was maintained at approximately 10–15 lux using a lux meter. The lighting schedule was maintained consistently, with no changes in photoperiod during the experimental period.

Environmental conditions were monitored daily. Ventilation was provided by natural open-sided housing that allowed air exchange through side openings; however, ventilation rate was not quantified during the study. All animal procedures were conducted in accordance with research ethics principles and animal welfare standards.

### Probiotic supplementation through drinking water

The probiotic used in this study was *B. amyloliquefaciens* strain FNCC 0079 (Waretha; Faculty of Animal Science, Universitas Andalas, Padang, Indonesia) in powder form. The probiotic product contained 1 × 10^10^ colony-forming units (CFUs)/g at the time of preparation.

### Probiotic administration

The probiotic was administered through drinking water throughout the experimental period. Fresh probiotic solution was prepared daily in the morning by dissolving the probiotic product in clean drinking water to achieve a final concentration of 1 × 10^8^ CFU/mL. Based on the product viability (1 × 10^10^ CFU/g), this concentration was achieved by dissolving 10 g of the probiotic in 1 L of drinking water (1% w/v). The probiotic solution was then provided ad libitum to all treatment groups each day during the study. The probiotic was administered to all treatments; therefore, the dietary factor evaluated was the inclusion level of the HI–PCL meal blend under a probiotic-supplemented feeding system.

### Daily preparation procedure

The probiotic solution was prepared daily using drinking water at room temperature (25°C–28°C). The probiotic powder was accurately weighed using an analytical balance at a rate of 10 g/L of drinking water. The powder was first dissolved in a small volume of clean water (approximately 200–300 mL) to facilitate homogenization and then diluted with additional water to reach the desired final volume. The solution was stirred for approximately 1–2 min until homogeneous before being offered to the ducks.

To maintain probiotic viability, the solution was temporarily stored in a closed or dark-colored container and protected from direct sunlight before administration. Drinkers were thoroughly cleaned before probiotic delivery, and disinfectants were not used during the supplementation period. Any remaining probiotic solution was discarded after 6 h of administration. This supplementation protocol was consistently applied throughout the 6-week experimental period.

### Probiotic viability verification

Probiotic viability at the beginning and end of the experiment was verified using the serial dilution plate-count method to confirm the number of viable bacteria in the product (CFU/g) and in the drinking water solution (CFU/mL). In the present study, the expected concentration ranged from 10^9^ to 10^10^ CFU/g for the product and from 10^7^ to 10^8^ CFU/mL for the drinking water solution, depending on mixing conditions and environmental exposure.

### Water intake

Water intake was not measured quantitatively for each treatment during the experiment. Therefore, the exact probiotic dose consumed at the individual level could not be calculated precisely. However, to minimize variation in probiotic exposure, the solution was prepared at the same concentration each day, provided at the same time daily, and any remaining solution was discarded after 6 h of administration.

### Experimental diets

The experimental diets consisted of different inclusion levels of the HI–PCL meal blend, namely 0% (control), 5%, 10%, and 15%, in the diets of 25-week-old laying Mojosari ducks during the 6-week rearing period. All diets were formulated to be iso-nitrogenous and iso-caloric, targeting approximately 17% crude protein and 2,700 kcal/kg metabolizable energy based on the proximate composition of each feed ingredient.

These nutrient targets were established according to the nutritional requirements of laying ducks during the early-to-mid laying phase. Diets for laying ducks generally contain approximately 16%–18% crude protein with metabolizable energy ranging from 2,650 to 2,750 kcal/kg to support optimal egg production performance [25]. Several studies conducted under tropical conditions have also indicated that diets within this nutrient range can maintain good laying performance at 25–31 weeks of age, corresponding to the early laying phase, when production is approaching peak.

The basal diet was formulated using yellow corn, rice bran, soybean meal, fish meal, coconut oil, vitamin–mineral premix, limestone, and the HI–PCL meal blend. The complete ingredient composition and inclusion levels are presented in Table 1, whereas the nutrient composition of the experimental diets is presented in Tables 2 and 3.

**Table 1:** Nutrient composition of the feed ingredients.

Feed ingredients (%)	Crude protein	Crude fat	Crude fiber	Calcium	Phosphorus	Metabolizable energy (kcal/kg)
Yellow corn^a^	9.87	3.98	4.06	0.11	0.38	3370
Rice bran^a^	10.63	12.40	15.60	0.83	1.97	1630
Soybean meal^a^	50.19	2.94	3.82	0.77	0.79	2240
Fish meal^a^	47.53	2.80	3.89	6.32	3.27	3080
BSF–SM meal blend^a^	48.15	14.77	7.61	5.47	1.48	3008
Coconut oil^b^	0	100	0	0	0	8600
Mineral Mix^c^	0	0	0	32.50	1.00	0
Limestone^a^	0	0	0	38.03	0.17	0

a Research analysis results, 2024,

b Scott *et al*. [28],

c Packaging label of PT Medion Indonesia

**Table 2:** Ingredient composition of each experimental diet.

Feed ingredients	A	B	C	D
Yellow corn	56	56	56	56
Rice bran	15	15	15	15
Soybean meal	10	10	10	6
Fish meal	11	6	1	0
BSF–SM meal blend	0	5	10	15
Coconut oil	0.5	0.5	0.5	0.5
Mineral mix	3.5	3.5	3.5	3.5
Limestone	4	4	4	4
Total	100	100	100	100

**Table 3:** Nutrient composition of the experimental diets.

Nutrient composition	A	B	C	D
Crude protein (%)	17.37	17.4	17.43	17.36
Crude fat (%)	5.19	5.79	6.39	6.98
Crude fiber (%)	5.42	5.61	5.8	5.98
Calcium (%)	3.62	3.57	3.53	3.71
Phosphorus (%)	0.99	0.9	0.81	0.82
Metabolizable energy (kcal/kg)	2737.5	2733.9	2730.3	2760.3

Values were calculated based on Tables 1 and 2.

### Diet mixing procedure and sampling

All feed ingredients were weighed according to the diet formulation and mixed using a horizontal feed mixer with a capacity of 50 kg per batch. Major ingredients were first added to the mixer and blended for 10 min; minor ingredients, such as the vitamin–mineral premix and coconut oil, were then added, and mixing continued for an additional 5 min until a homogeneous mixture was obtained. Each batch of feed weighed approximately 50 kg.

For nutrient composition analysis of the experimental diets, samples were collected using a multiple-point sampling method. Five subsamples were randomly collected from different locations within each mixed batch, then pooled and homogenized to produce a composite sample for each dietary treatment. Composite samples were analyzed for crude protein, crude fat, crude fiber, calcium, total phosphorus, and gross energy [26]. Metabolizable energy was calculated from gross energy and nutrient composition using standard poultry nutrition evaluation procedures.

### Proximate analysis of HI and PCL ingredients

Before diet formulation, the HI and PCL meals were analyzed separately to determine crude protein, crude fat, crude fiber, ash, moisture content, calcium, phosphorus, metabolizable energy, and chitin content in the HI meal. The analysis showed that the HI meal contained crude protein 52%, crude fat 17%, crude fiber 9%, calcium 6.60%, phosphorus 1.48%, metabolizable energy 3,300 kcal/kg, and chitin content 24%. In contrast, the PCL meal contained crude protein 25%, crude fat 5%, crude fiber 2%, calcium 1.20%, phosphorus 2.40%, and metabolizable energy 2,200 kcal/kg. The HI–PCL meal blend (80% HI and 20% PCL) contained crude protein 48.15%, crude fat 14.77%, crude fiber 7.61%, calcium 5.47%, phosphorus 1.10%, metabolizable energy 3,008 kcal/kg, and chitin content 19.26%.

The 80:20 HI-to-PCL ratio was selected to combine the nutritional advantages of both local feed resources. HI meal is recognized as a rich source of protein and fat, whereas PCL meal provides relatively high mineral content, particularly calcium. This combination was expected to achieve a more balanced nutrient profile to support protein and mineral requirements in laying ducks. Furthermore, this proportion was selected to avoid excessively high chitin levels from the insect-based ingredient, which may negatively affect nutrient digestibility at higher inclusion levels.

### Proximate analysis and chitin determination

Proximate analysis was conducted following standard methods of the Association of Official Analytical Chemists. Moisture content was determined using method 934.01, ash using method 942.05, crude protein using the Kjeldahl method (984.13), crude fat using Soxhlet extraction (920.39), and crude fiber using method 978.10. Calcium and phosphorus were determined according to methods 968.08 and 965.17, respectively. Gross energy was analyzed using a bomb calorimeter [27], whereas metabolizable energy was estimated using prediction equations based on proximate composition commonly applied in poultry nutrition studies.

Chitin content of HI meal and the HI–PCL meal blend was determined using a gravimetric method involving demineralization and deproteinization. Samples were first demineralized with 1 M HCl, then deproteinized with 1 M NaOH. The remaining residue was washed, dried at 60°C until constant weight, and expressed as percentage chitin content.

### Particle size after grinding and sieving

HI meal, PCL meal, and the HI–PCL meal blend were ground and sieved using a 60-mesh screen (approximately 250 μm), resulting in relatively uniform particle size with an average diameter of approximately 200–300 μm. This uniformity was intended to minimize feed selection by ducks and ensure homogeneous mixing of the experimental diets.

### Storage conditions of ingredients before diet mixing

Before diet mixing, the HI meal, the PCL meal, and the HI–PCL meal blend were stored in tightly sealed plastic packaging and maintained in a dry storage room at approximately 25 ± 2°C with relative humidity of 60%–70% for up to 2 weeks. The ingredients were stored away from direct light to maintain quality stability, prevent fat oxidation, and minimize microbial contamination before diet formulation.

All laying Mojosari ducks in each treatment received *B. amyloliquefaciens* supplementation through drinking water according to the supplementation protocol described above to support nutrient utilization and maintain consistent treatment conditions throughout the experimental period.

### Parameters and analytical methods

**Feed intake:** Feed intake was calculated as the difference between the amount of feed offered and the amount refused in each experimental unit during the observation period. Feed was weighed using a digital scale (SF-400 Digital Scale; Zhejiang, China) with an accuracy of ±0.01 g before being offered to the ducks. At the end of the observation period, feed refusals were collected and reweighed using the same scale. Feed intake was calculated as the difference between feed offered and feed refused.

**Daily egg production:** Eggs were collected twice daily, in the morning (08:00 WIB) and afternoon (16:00 WIB), to minimize the risk of egg breakage or loss. Daily egg production was calculated as the percentage of eggs produced in each experimental unit relative to the number of ducks housed in that unit on the day of observation.

**Egg weight:** Egg weight was determined by weighing each egg produced from each experimental unit using a precision digital balance (AND EK-300i; A&D Company Ltd., Tokyo, Japan) with an accuracy of ±0.01 g. Weighing was performed immediately after egg collection to minimize weight changes due to moisture loss.

**Egg mass:** Egg mass was calculated to represent total egg output per bird per day (g/bird/day). Egg mass was obtained by multiplying daily egg production (%) by the average egg weight (g) in each experimental unit.

**Feed conversion ratio (FCR):** FCR was calculated by dividing feed intake by egg mass for each experimental unit during the observation period using the following equation:

FCR = feed intake (g) / egg mass (g)

A lower FCR indicates better feed efficiency for egg production.

Egg quality of Mojosari duck eggs: Eggs for quality analysis were collected at the end of each rearing week. After collection, eggs were stored at controlled room temperature (25 ± 2°C) for no longer than 24 h before egg quality assessment. Cracked, broken, or double-yolk eggs were excluded from egg quality analysis. The proportion of excluded eggs was less than 5% of the total egg samples in each treatment.

**Yolk index:** Yolk index was calculated as the ratio of yolk height to the average yolk diameter. Yolk height was measured using a digital caliper (Mitutoyo Corporation, Kanagawa, Japan) with an accuracy of ±0.01 mm, whereas yolk diameter was measured in two perpendicular directions and averaged.

Yolk index = yolk height (mm) / average yolk diameter (mm)

**Haugh unit (HU):** HU is a standard indicator of internal egg quality, particularly albumen freshness. Albumen height was measured using a tripod micrometer (AMES S-6428; B.C. Ames Company, Waltham, MA, USA) with an accuracy of ±0.01 mm, whereas egg weight was measured using a precision digital balance. HU was calculated using the following equation:

HU = 100 × log_10_ (H − 1.7W^0.37 + 7.6)

where H is albumen height (mm) and W is egg weight (g).

**Yolk color:** Yolk color was evaluated using the DSM YolkFan™ (DSM Nutritional Products, Basel, Switzerland) with a color scale ranging from 1 to 15, where higher values indicated greater yolk color intensity. Scoring was performed visually by comparing fresh yolk color with the reference standards on the DSM YolkFan™.

Observations were conducted under standardized white fluorescent lighting with a color temperature of approximately 5,500 K to ensure consistent color perception. Before evaluation, the DSM YolkFan™ was checked to confirm that the reference colors remained clear and unchanged. Each egg sample was independently assessed by two trained evaluators. To reduce subjectivity, both evaluators performed visual calibration using several standard yolk examples before scoring. The final yolk color value was obtained by averaging the scores from the two evaluators.

**Egg length:** Egg length (distance from the blunt end to the pointed end) was measured using a digital caliper (Mitutoyo Corporation) with an accuracy of ±0.01 mm.

**Egg width:** Egg width was measured at the equatorial region of the egg using the same digital caliper with an accuracy of ±0.01 mm.

**Income over feed cost (IOFC):** IOFC was calculated to evaluate the economic efficiency of the experimental diets. IOFC was determined as the difference between income from egg sales and total feed cost during the study period. Income was calculated as total egg production multiplied by the farm-gate egg price at the time of the study, whereas feed cost was calculated as total feed intake multiplied by the diet price for each treatment.

**Preparation procedure for the HI–PCL meal blend**

**Preparation of *H. illucens* meal:** *H. illucens* larvae were harvested at the late larval stage (approximately 15–17 days) and washed under running water to remove residual substrate. The larvae were then blanched at 90°C–100°C for 3–5 min to reduce microbial contamination and inactivate enzymatic activity. After blanching, the larvae were drained and dried in a drying oven at 55°C–60°C for 24–48 h or until moisture content reached ≤10%. The dried larvae were ground into a fine meal and sieved to obtain a uniform particle size before storage in airtight containers.

**Preparation of *P. canaliculata* meal:** *P. canaliculata* was collected from paddy field areas and thoroughly washed under running water to remove mud and debris. The snails were then boiled at 95°C–100°C for 10–15 min to facilitate separation of the flesh from the shell and reduce microbial contamination. The flesh was separated from the shells and cut into small pieces to accelerate drying. The snail meat was dried in an oven at 55°C–60°C for 48–72 h or until the moisture content reached ≤10%, then ground into meal and sieved to obtain a uniform particle size. The PCL meal was stored in airtight containers before mixing.

**Preparation of the HI–PCL meal blend (80:20):** The HI–PCL meal blend was prepared by mixing HI meal and PCL meal at an 80:20 ratio (w/w). Mixing was performed using a feed mixer for approximately 10–15 min until a homogeneous blend was obtained. The homogenized blend was stored in airtight containers in a dry storage area at room temperature until incorporation into the experimental diets.

**Feed quality control:** To ensure ingredient consistency, moisture content (≤10%) was measured and proximate analysis was performed for HI meal, PCL meal, and the HI–PCL meal blend before diet formulation.

### Statistical analysis

All data were analyzed using analysis of variance with a completely randomized design, with four dietary treatments corresponding to different inclusion levels of the HI–PCL meal blend (0%, 5%, 10%, and 15%). Statistical analyses were performed using IBM SPSS Statistics version 27 (IBM Corp., Armonk, NY, USA).

In this study, the experimental unit was the pen, with each pen housing 10 laying Mojosari ducks. The mean value for each pen was treated as a single observation in the statistical analysis.

Before conducting analysis of variance, the data were tested for statistical assumptions, including normality using the Shapiro–Wilk test and homogeneity of variances using Levene’s test.

The statistical model used in this study was as follows:

Yij = μ + τi + εij

where:

Yij = observed value for the ith treatment and jth replicate

μ = overall mean

τi = effect of the ith treatment

εij = experimental error

If the analysis of variance showed significant differences among treatments (p < 0.05), Duncan’s Multiple Range Test [29] was used as a post hoc test to compare treatment means. All statistical tests were performed at a significance level of α = 0.05. Exact p-values were reported in tables or text to provide more detailed information regarding the level of significance among treatments.

## RESULTS AND DISCUSSION

### Production performance of laying Mojosari ducks

The performance of 25-week-old laying Mojosari ducks fed diets with different inclusion levels of the HI–PCL meal blend (0%, 5%, 10%, and 15%) and supplemented with the probiotic *B. amyloliquefaciens* for 6 weeks is presented in Table 4.

**Table 4:** Performance of 25-week-old laying Mojosari ducks at different inclusion levels of the HI–PCL meal blend with *Bacillus amyloliquefaciens* supplementation (6-week trial).

Treatment	Feed intake (g/bird/day)	Daily egg production (%)	Egg weight (g/egg)	Egg mass (g/bird/day)	Feed conversion ratio
A	141.05 ± 1.87ᵃ	61.90% ± 0.05ᵃ	64.69 ± 0.65ᶜ	39.75 ± 3.11ᵃ	3.78 ± 0.31ᵃ
B	149.18 ± 1.59ᵇ	70.95% ± 0.05ᵇ	66.18 ± 0.69ᵇ	46.59 ± 3.09ᵇ	3.34 ± 0.19ᵇ
C	149.03 ± 0.49ᵇ	70.76% ± 0.06ᵇ	67.10 ± 0.35ᵇ	47.40 ± 3.62ᵇ	3.29 ± 0.21ᵇ
D	147.94 ± 0.91ᵇ	72.38% ± 0.06ᵇ	68.96 ± 1.61ᵃ	49.66 ± 3.35ᵇ	3.14 ± 0.21ᵇ
SE	0.6	0.03	0.43	1.47	0.1
p-value	*	*	*	*	*

A: 0% HI–PCL meal blend, B: 5% HI–PCL meal blend, C: 10% HI–PCL meal blend, D: 15% HI–PCL meal blend, SE: Standard error ᵃ⁻ᵇDifferent superscript letters in the same column indicate significant differences (p < 0.05)

**Feed intake:** Feed intake of 25-week-old laying Mojosari ducks fed diets with different inclusion levels of the HI–PCL meal blend (0%, 5%, 10%, and 15%) with *B. amyloliquefaciens* supplementation ranged from 141.05 ± 1.87 to 149.18 ± 1.59 g/bird/day. Treatment A (0% HI–PCL meal blend) resulted in the lowest feed intake (141.05 ± 1.87 g/bird/day), whereas treatments B (5%), C (10%), and D (15%) showed feed intake of 149.18 ± 1.59, 149.03 ± 0.49, and 147.94 ± 0.91 g/bird/day, respectively. Analysis of variance indicated that HI–PCL meal blend inclusion level significantly affected feed intake (p < 0.05). Duncan’s Multiple Range Test showed that feed intake in treatment A differed significantly (p < 0.05) from treatments B, C, and D, whereas treatment B did not differ (p > 0.05) from treatments C and D.

The higher feed intake observed in the HI–PCL-containing diets (5%–15%) may be associated with changes in dietary characteristics affecting palatability and physiological intake responses. Insect-based ingredients derived from *H. illucens* contain specific fat fractions and fatty acids, including lauric acid, that can modify the aroma and physical properties of the diet. Therefore, at certain inclusion levels, they may not suppress intake and may even stimulate consumption when dietary energy–protein balance is maintained. The use of *H. illucens* at appropriate levels can be sustained without reducing feed intake or performance, depending on ingredient form and overall diet composition [30]. The fatty acid profile of *H. illucens* reported includes lauric acid 32.25%, myristic acid 16.57%, palmitic acid 16.97%, stearic acid 4.13%, and capric acid 0.20% [31]. Increased feed intake may also relate to dietary glutamic acid content, because higher glutamic acid levels have been associated with increased feed consumption in laying Mojosari ducks, likely because of improved palatability [32]. The amino acid composition reported for *H. illucens* includes glutamic acid 1.40%, serine 0.80%, aspartic acid 0.70%, lysine 0.30%, and methionine 0.30% [33].

The higher feed intake in treatments B and C suggests that HI–PCL meal blend inclusion at 5%–10% remained well accepted by laying ducks. At these levels, chitin from the insect ingredient may not have been sufficiently high to disrupt digestion or suppress appetite. Although chitin can reduce protein digestibility when insect ingredients are included at excessive levels, its effects may be minimal at 5%–10% because poultry can still adapt. Moreover, because the diets were formulated to be iso-nitrogenous and iso-caloric across treatments, nutrient requirements were maintained, allowing normal and stable feed intake [19]. Supplementation with *B. amyloliquefaciens* via drinking water may also have helped maintain gastrointestinal comfort and nutrient utilization, thereby preventing reductions in feed intake. *Bacillus*-based probiotics are known to produce digestive enzymes such as proteases and amylases and to modulate gut microbiota, thereby supporting digestion and improving feed utilization, which is particularly relevant for non-conventional diets with more complex nutrient fractions [31].

**Daily egg production:** Daily egg production of 25-week-old laying Mojosari ducks fed different inclusion levels of the HI–PCL meal blend (0%, 5%, 10%, and 15%) with *B. amyloliquefaciens* supplementation ranged from 61.90 ± 0.05% to 72.38 ± 0.06%. Treatment A (0% HI–PCL meal blend) produced the lowest daily egg production (61.90 ± 0.05%), whereas treatments B (5%), C (10%), and D (15%) produced 70.95 ± 0.05%, 70.76 ± 0.06%, and 72.38 ± 0.06%, respectively. Analysis of variance showed that the inclusion level of the HI–PCL meal blend significantly affected daily egg production (p < 0.05). Duncan’s Multiple Range Test indicated that treatment A differed significantly (p < 0.05) from treatments B, C, and D, whereas treatment B did not differ (p > 0.05) from treatments C and D.

The increase in egg production in treatments B–D indicates that the combined alternative protein sources (*H. illucens* and *P. canaliculata*) were able to meet the nutrient requirements of laying ducks, particularly protein and amino acids needed for egg formation. *H. illucens* is recognized for high protein content and a favorable amino acid profile, and at certain inclusion levels, it can improve laying performance and several egg quality traits, although responses depend on inclusion level and ingredient form [35]. Supplementation with *H. illucens* has been linked to increased daily egg production and improved production efficiency, supporting its potential role in sustaining output in waterfowl [36]. *P. canaliculata* may serve as an alternative feed ingredient because of its protein content and functional components, complementing insect-based ingredients and improving overall diet quality [37]. Probiotic supplementation with *B. amyloliquefaciens* via drinking water may further enhance nutrient utilization, particularly when diets contain non-conventional ingredients with variable digestibility. *B. amyloliquefaciens* is a spore-forming bacterium capable of producing digestive enzymes and modulating gut microbiota, thereby improving nutrient availability for egg production. In laying hens, *B. amyloliquefaciens* supplementation has been reported to improve performance and egg quality by enhancing gut conditions and microbiota balance [24].

**Egg weight:** Egg weight of 25-week-old laying Mojosari ducks fed different inclusion levels of the HI–PCL meal blend with *B. amyloliquefaciens* supplementation ranged from 64.69 ± 0.65 to 68.96 ± 1.61 g/egg. Treatment A (0% HI–PCL meal blend) had the lowest egg weight (64.69 ± 0.65 g/egg), whereas treatments B (5%), C (10%), and D (15%) showed egg weights of 66.18 ± 0.69, 67.10 ± 0.35, and 68.96 ± 1.61 g/egg, respectively. Analysis of variance indicated a significant effect of HI–PCL inclusion level on egg weight (p < 0.05). Duncan’s Multiple Range Test showed that egg weight in treatment A differed significantly (p < 0.05) from treatments B, C, and D; treatment B did not differ (p > 0.05) from treatment C; and treatments B and C differed significantly (p < 0.05) from treatment D.

Higher egg weight in the HI–PCL-containing treatments suggests that the blend supplied amino acids supporting egg component deposition, particularly lysine and methionine. *H. illucens* has high protein content and a competitive amino acid profile, and at appropriate inclusion levels, it may improve laying performance and egg production traits when diets are well balanced [38]. Egg weight is strongly influenced by the adequacy of essential amino acids, especially methionine and lysine, which play key roles in albumen synthesis and egg protein deposition. Methionine supplementation has been shown to affect egg weight and the albumen-to-yolk ratio and improve protein utilization efficiency in laying hens [39]. Lysine is also an essential nutrient influencing layer performance [40]. Reported amino acid contents for *H. illucens* include lysine 0.30% and methionine 0.30% [33]. In addition, *P. canaliculata* meat meal contains relatively high levels of essential amino acids, with methionine at 1.07% and lysine at 3.26% on an air-dry basis [41], which may help support egg formation when included in the blend.

**Egg mass:** Egg mass of 25-week-old laying Mojosari ducks fed different inclusion levels of the HI–PCL meal blend with *B. amyloliquefaciens* supplementation ranged from 39.75 ± 3.11 to 49.66 ± 3.35 g/bird/day. Treatment A (0% HI–PCL meal blend) produced the lowest egg mass (39.75 ± 3.11 g/bird/day), whereas treatments B (5%), C (10%), and D (15%) produced 46.59 ± 3.09, 47.40 ± 3.62, and 49.66 ± 3.35 g/bird/day, respectively. Analysis of variance showed a significant effect of HI–PCL inclusion level on egg mass (p < 0.05). Duncan’s Multiple Range Test indicated that treatment A differed significantly (p < 0.05) from treatments B, C, and D, whereas treatment B did not differ (p > 0.05) from treatments C and D.

The increase in egg mass in treatments B–D is expected because egg mass integrates daily egg production and egg weight; therefore, improvements in both components increase total egg output. In the present study, inclusion of the HI–PCL meal blend increased both daily egg production and egg weight compared with the control, consistent with reports that *H. illucens* is protein-rich and has a competitive amino acid profile that can support laying performance when diets are properly balanced [35]. The observation that egg mass did not always differ significantly among 5%–15% inclusion levels suggests a tendency toward a plateau response once major nutrient requirements are met, as also reported in laying poultry fed *H. illucens*-based diets where responses are not necessarily linear [14]. Supplementation with *B. amyloliquefaciens* may have enhanced nutrient utilization by improvnging intestinal integrity and modulation the gut microbiota, thereby supporting egg production efficiency in non-conventional diets [24]. Overall, these findings indicate that inclusion of an HI–PCL meal blend at 5%–15%, supported by probiotic supplementation, can increase egg mass output in laying Mojosari ducks when diets are formulated to be iso-nitrogenous and iso-caloric.

**FCR:** Feed conversion ratio of 25-week-old laying Mojosari ducks fed different inclusion levels of the HI–PCL meal blend with *B. amyloliquefaciens* supplementation ranged from 3.14 ± 0.21 to 3.78 ± 0.31. Treatment A (0% HI–PCL meal blend) showed the highest FCR (3.78 ± 0.31), whereas treatments B (5%), C (10%), and D (15%) showed FCR values of 3.34 ± 0.19, 3.29 ± 0.21, and 3.14 ± 0.21, respectively. Analysis of variance indicated that HI–PCL inclusion level significantly affected FCR (p < 0.05). Duncan’s Multiple Range Test showed that treatment A differed significantly (p < 0.05) from treatments B, C, and D, whereas treatment B did not differ (p > 0.05) from treatments C and D.

The improved FCR in treatments B–D indicates enhanced feed efficiency, as feed intake resulted in higher egg mass compared with the control. In general, FCR improves when egg production and egg weight increase without a proportional increase in feed intake, reflecting more efficient conversion of nutrients into egg mass. The use of *H. illucens* in laying poultry can improve performance and feed efficiency depending on inclusion level and product form [35]. *P. canaliculata* may complement diet quality through its protein and mineral content; however, standardization of the ingredient and appropriate formulation are necessary to achieve consistent outcomes [37]. Furthermore, *B. amyloliquefaciens* supplementation has been reported to enhance nutrient utilization efficiency in poultry under certain conditions [42].

Overall, the relationships among production performance parameters reflect physiological linkages between feed intake, nutrient utilization, and egg mass formation. Increased feed intake in the HI–PCL meal blend treatments indicates that the diets remained palatable and provided sufficient nutrient supply to support egg production. Higher nutrient intake may increase the availability of energy and amino acids for the synthesis of egg components, particularly albumen and yolk, thereby contributing to higher egg mass. Although structural components such as chitin in *H. illucens* may reduce nutrient digestibility when included at high levels, the inclusion range used in this study (5%–15%) did not appear to adversely affect production performance. This outcome may also have been supported by *B. amyloliquefaciens* supplementation, which can enhance digestive enzyme activity and modulate gut microbiota, helping maintain efficient nutrient utilization in diets based on alternative feed ingredients. Thus, HI–PCL meal blend at moderate inclusion levels can support optimal laying performance in Mojosari ducks when diets are formulated to be iso-nitrogenous and iso-caloric. Internal egg quality of 25-week-old Mojosari duck eggs at different inclusion levels of the HI–PCL meal blend with Bacillus amyloliquefaciens supplementation (6-week trial) is presented in Table 5.

**Table 5:** Internal egg quality of 25-week-old Mojosari duck eggs at different inclusion levels of the HI–PCL meal blend with *Bacillus amyloliquefaciens* supplementation (6-week trial).

Treatment	Yolk index	Haugh unit	Yolk color
A	0.41 ± 0.02ᵃ	75.14 ± 5.50ᵃ	9.44 ± 0.29ᵃᵇ
B	0.38 ± 0.01ᵇ	69.84 ± 4.65ᵇ	9.13 ± 0.54ᵇ
C	0.40 ± 0.01ᵃ	70.94 ± 3.30ᵇ	9.21 ± 0.81ᵇ
D	0.38 ± 0.02ᵃ	67.54 ± 3.35ᵇ	10.11 ± 0.21ᵃ
SE	0.007	1.94	0.23
P-value	*	*	*

A: 0% HI–PCL meal blend, B: 5% HI–PCL meal blend, C: 10% HI–PCL meal blend, D: 15% HI–PCL meal blend, SE: Standard error

* : Significant differences (p < 0.05)

ᵃ⁻ᵇDifferent superscript letters in the same column indicate significant differences (p < 0.05).

### Internal egg quality of Mojosari duck eggs

**Yolk index:** The yolk index of 25-week-old laying Mojosari ducks fed different inclusion levels of the HI–PCL meal blend (0%, 5%, 10%, and 15%) with *B. amyloliquefaciens* supplementation ranged from 0.38 ± 0.01 to 0.41 ± 0.02. Treatment A (0% HI–PCL meal blend) showed the highest yolk index (0.41 ± 0.02), whereas treatments B (5%), C (10%), and D (15%) showed yolk index values of 0.38 ± 0.01, 0.40 ± 0.01, and 0.38 ± 0.02, respectively. Analysis of variance indicated that HI–PCL meal blend inclusion level significantly affected yolk index (p < 0.05). Duncan’s Multiple Range Test showed that yolk index in treatment A differed significantly (p < 0.05) from treatment B, but did not differ (p > 0.05) from treatments C and D.

Yolk index is influenced by the strength of the vitelline membrane and by the balance of water, lipid, and protein fractions in the yolk, which determine yolk firmness after the egg is broken. The changes in yolk index observed with HI–PCL inclusion may be related to alterations in dietary lipid composition and structural components of alternative ingredients. *H. illucens* contains specific lipid fractions and chitin that may affect digestion and lipid metabolism, potentially influencing nutrient deposition into the egg. This is consistent with reports that *H. illucens* can modify yolk fatty acid profiles in laying poultry [43]. Dietary lipid composition is known to influence egg characteristics in waterfowl [44]. The effect of *B. amyloliquefaciens* on yolk index is likely indirect, because probiotics primarily influence nutrient utilization and some egg quality traits, whereas effects on yolk index are not always consistent across studies [45].

**HU:** HU values of Mojosari duck eggs from treatments containing different HI–PCL meal blend levels with *B. amyloliquefaciens* supplementation showed that treatment A produced the highest HU (75.14 ± 5.50), whereas treatments B (5%), C (10%), and D (15%) resulted in HU values of 69.84 ± 4.65, 70.94 ± 3.30, and 67.54 ± 3.35, respectively. Analysis of variance demonstrated that the inclusion level of the HI–PCL meal blend significantly affected HU (p < 0.05). Based on Duncan’s Multiple Range Test, HU in treatment A differed significantly (p < 0.05) from treatments B, C, and D, whereas treatment B did not differ (p > 0.05) from treatments C and D.

HU is strongly influenced by the height of the thick albumen and albumen viscosity; therefore, it is sensitive to nutrient balance and to protein–energy metabolism associated with albumen protein deposition. The reduction in HU observed in the HI–PCL treatments may be related to structural components, such as chitin, and the lipid profile of *H. illucens*, which can affect nutrient digestibility and metabolism. In waterfowl, insect-based ingredients such as *H. illucens* may improve production performance but do not necessarily uniformly enhance all egg quality parameters, as albumen formation is influenced by complex nutritional and physiological factors [36]. Nevertheless, HU values across all treatments (67.54–75.14) remained within a good-to-very-good range for fresh eggs. In practical terms, HU values above approximately 60–70 are generally considered acceptable indicators of good internal egg quality, whereas values above 75 indicate very good quality. Thus, the decrease in HU observed in HI–PCL treatments is unlikely to have a substantial negative impact on the eggs’ shelf life, marketability, or consumer acceptance.

In addition, yolk index values in the present study ranged from 0.38 to 0.41 and remained within the normal range for fresh eggs (approximately 0.35–0.42), indicating that yolk structural integrity and freshness were maintained across treatments. *B. amyloliquefaciens* supplementation may have contributed to maintaining gut health and improving nutrient utilization, helping alternative feed ingredients sustain overall egg quality and production performance [46].

**Yolk color:** Yolk color scores of 25-week-old laying Mojosari ducks fed different HI–PCL meal blend inclusion levels (0%, 5%, 10%, and 15%) with *B. amyloliquefaciens* supplementation ranged from 9.13 ± 0.54 to 10.11 ± 0.21. Treatment D (15% HI–PCL meal blend) produced the highest yolk color score (10.11 ± 0.21), whereas treatments C (10%), B (5%), and A (0%) showed values of 9.21 ± 0.81, 9.13 ± 0.54, and 9.44 ± 0.29, respectively. Analysis of variance indicated that HI–PCL meal blend inclusion level significantly affected yolk color (p < 0.05). Duncan’s Multiple Range Test showed that yolk color in treatment D differed significantly (p < 0.05) from treatments C, B, and A. Treatments B and C did not differ significantly (p > 0.05) from treatment A, but both differed significantly (p < 0.05) from treatment D.

Yolk color is primarily determined by the deposition of carotenoid pigments (xanthophylls) derived from the diet, because poultry cannot synthesize carotenoids endogenously. Therefore, yolk color intensity depends on pigment availability in the diet and absorption efficiency in the gastrointestinal tract. The increased yolk color score at 15% HI–PCL inclusion may be related to nutritional characteristics of the blend, particularly the relatively high lipid fraction of *H. illucens*. Dietary lipids can enhance the absorption of fat-soluble nutrients, including carotenoids, thereby increasing pigment deposition into the yolk. Moreover, previous studies have suggested that *H. illucens* meal may influence lipid metabolism and nutrient deposition into eggs, potentially modifying yolk color intensity. The inclusion of *P. canaliculata* meal may also provide nutrients, including protein and minerals, that support metabolism and egg formation. Probiotic supplementation with *B. amyloliquefaciens* may have further strengthened these effects by improving gut health and nutrient utilization efficiency, thereby enhancing pigment absorption and deposition.

However, carotenoid content was not directly analyzed in the diets or yolks in this study. Therefore, the mechanism underlying the increased yolk color at higher HI–PCL inclusion levels remains a physiological hypothesis, namely improved absorption of fat-soluble pigments. Further studies measuring carotenoid concentrations in diets and yolks are needed to confirm this mechanism. Practically, the yolk color scores obtained in this study (approximately 9–10) represent relatively intense yolk coloration based on standard scales such as the Roche/DSM Yolk Color Fan. Yolk color is an important visual quality attribute for consumers because deeper yolk coloration is often associated with better nutritional quality and product appeal. This is particularly relevant to duck eggs, commonly used in processed products such as salted eggs, where consumers generally prefer eggs with more intense yolk color. Thus, the increase in yolk color score to approximately 10 in the 15% HI–PCL treatment is not only statistically significant but may also provide added value in terms of visual attractiveness and consumer acceptance of the eggs.

**Egg length:** Egg length of 25-week-old laying Mojosari ducks fed diets with different inclusion levels of the HI–PCL meal blend (0%, 5%, 10%, and 15%) with *B. amyloliquefaciens* supplementation ranged from 51.07 ± 0.75 to 54.42 ± 3.69 mm. Treatment A (0% HI–PCL meal blend) produced an egg length of 51.07 ± 0.75 mm, treatment B (5%) 51.07 ± 1.42 mm, treatment C (10%) 51.93 ± 0.88 mm, and treatment D (15%) 54.42 ± 3.69 mm. Analysis of variance showed that HI–PCL meal blend inclusion level did not affect egg length (p > 0.05).

Egg length is a morphometric trait reflecting egg size and shape and is generally influenced by genetic factors, bird age, and dietary nutrient balance. The present results indicate that inclusion of the HI–PCL meal blend up to 15%, combined with *B. amyloliquefaciens* supplementation, did not significantly affect egg length in laying Mojosari ducks. This suggests that the use of these alternative ingredients did not disrupt egg formation or the development of structural characteristics that determine egg dimensions. The lack of significant differences in egg length among treatments also suggests that nutrient availability across diets was relatively comparable, particularly for protein, energy, and minerals involved in egg formation. Egg size is largely a breed-related trait, whereas nutritional manipulation more commonly influences albumen deposition, shell strength, and cracking incidence rather than egg dimensions [47]. Similarly, formulating local diets with comparable protein and energy levels in Fayoumi chickens produced significant differences in egg production and egg weight, whereas shell traits and egg shape index were not markedly altered across diets [48]. Therefore, inclusion of the HI–PCL meal blend as an alternative protein source did not negatively affect egg physical characteristics, particularly egg length. External egg quality of 25-week-old Mojosari duck eggs at different inclusion levels of the HI–PCL meal blend with Bacillus amyloliquefaciens supplementation (6-week trial) is presented in Table 6.

**Table 6:** External egg quality of 25-week-old Mojosari duck eggs at different inclusion levels of the HI–PCL meal blend with *Bacillus amyloliquefaciens* supplementation (6-week trial).

Treatment	Egg length (mm)	Egg width (mm)
A	51.07 ± 0.75	27.52 ± 1.23
B	51.07 ± 1.42	27.41 ± 0.63
C	51.93 ± 0.88	27.91 ± 0.58
D	54.42 ± 3.69	27.35 ± 0.91
SE	1.19	0.51
P-value	NS	NS

A: 0% HI–PCL meal blend, B: 5% HI–PCL meal blend, C: 10% HI–PCL meal blend, D: 15% HI–PCL meal blend, SE: Standard error, NS: Non-significant.

### External egg quality of Mojosari duck eggs

**Egg width:** Egg width of 25-week-old laying Mojosari ducks fed different HI–PCL meal blend inclusion levels with *B. amyloliquefaciens* supplementation ranged from 27.35 ± 0.91 to 27.91 ± 0.58 mm. Treatment A (0% HI–PCL meal blend) resulted in an egg width of 27.52 ± 1.23 mm, treatment B (5%) 27.41 ± 0.63 mm, treatment C (10%) 27.91 ± 0.58 mm, and treatment D (15%) 27.35 ± 0.91 mm. Analysis of variance showed that HI–PCL meal blend inclusion level did not affect egg width (p > 0.05).

Egg width is another morphometric parameter describing overall egg dimensions and is closely related to egg shape. The results indicate that inclusion of the HI–PCL meal blend up to 15%, together with *B. amyloliquefaciens* supplementation, did not significantly influence egg width. This further supports the conclusion that the inclusion of alternative ingredients did not affect egg formation processes associated with physical egg dimensions. The absence of significant differences among treatments suggests that the diets’ nutrient balance was broadly similar, particularly for protein, energy, and minerals involved in egg formation. Comparative studies have shown that differences in egg traits such as egg weight, shell thickness, chemical composition, and yolk texture are often primarily driven by genotype rather than dietary differences alone [49]. Moreover, improvements in laying performance in modern poultry have largely been achieved through genetic selection, and egg characteristics are strongly influenced by genetics and breeder age [50]. Thus, the HI–PCL meal blend can be considered a viable alternative protein source in laying duck diets without adversely affecting egg physical dimensions, particularly egg length and width.

### Economic evaluation

In treatment A (0% HI–PCL/control), the IOFC of IDR 651,939.27 reflects the margin generated from the combination of egg income and feed costs. Because the egg selling price was the same (IDR 40,000/kg), differences in IOFC were primarily driven by egg production (income) and feed cost (feed intake × diet price). IOFC is commonly used as a simple indicator to evaluate the effect of feeding programs on profitability, representing the remaining income after deducting feed costs, before accounting for other costs such as labor, utilities, and health management.

Treatment D (15% HI–PCL) produced the highest IOFC (IDR 847,176.64) because two economic mechanisms operated simultaneously: income increased because of the highest egg output, and feed cost per kilogram of diet decreased because the diet price was the lowest, reflecting substitution of conventional protein ingredients. Biologically, this is consistent with reports that *H. illucens* is a promising protein and fat source with a nutritional profile that can approach major protein ingredients such as fish meal and soybean meal, thereby potentially reducing formulation costs without compromising performance when included at appropriate levels [9]. The use of *P. canaliculata* as a feed ingredient has also been reported to affect productive responses and composition in waterfowl, suggesting its potential as a complementary local protein source in diets [51]. The IOFC for each treatment at the end of the experiment (IDR/kg) is presented in Table 7.

**Table 7:** Income over feed cost for each treatment at the end of the experiment (IDR/kg).

Description	A	B	C	D
I. Income				
1. Egg production (kg)	84.09	86.03	87.23	89.64
2. Selling price (IDR/kg)	40000	40000	40000	40000

**Description**	**A**	**B**	**C**	**D**

Total income (IDR)	33,63,729.92	34,41,206.46	34,89,044.33	35,85,760.01
1. Feed intake (kg)	296.21	313.28	312.95	310.67
2. Feed price (IDR/kg)	9155	9055	8955	8815
Total expenditure (IDR)	27,11,790.65	28,36,792.05	28,02,502.17	27,38,583.38
III. Income over feed cost (IDR)	6,51,939.27	6,04,414.41	6,86,542.15	8,47,176.64

Overall, the comparison of treatments A and D indicates that a 15% inclusion of HI–PCL resulted in a higher feed margin because increased egg production raised total income, whereas the lower diet price in treatment D reduced feed costs, such that the increase in expenditure was not proportional to the increase in income. Consequently, IOFC in treatment D was higher than in the control group.

## CONCLUSION

The present study demonstrated that dietary inclusion of the HI–PCL meal blend (*H. illucens* and *P. canaliculata*) combined with *B. amyloliquefaciens* supplementation positively influenced the productive performance of laying Mojosari ducks. Inclusion levels of 5%–15% significantly improved feed intake, daily egg production, egg weight, egg mass, and FCR compared with the control diet (p < 0.05). The 15% HI–PCL treatment produced the highest daily egg production (72.38%), egg weight (68.96 g/egg), egg mass (49.66 g/bird/day), and the most efficient FCR (3.14). In addition, yolk color intensity increased significantly at the 15% inclusion level, whereas egg length and egg width were not adversely affected. Economically, the 15% HI–PCL treatment generated the highest IOFC (IDR 847,176.64), indicating improved profitability through increased egg output and lower feed costs.

These findings indicate that the HI–PCL meal blend can serve as a viable alternative protein source in laying duck diets without compromising egg quality or productive performance. The combination of insect- and snail-based ingredients offers an opportunity to reduce reliance on conventional protein sources such as soybean meal and fish meal, which are often associated with price volatility and sustainability concerns. Moreover, utilization of *H. illucens* and *P. canaliculata* supports environmentally sustainable livestock production by converting locally available biological resources into nutritionally valuable feed ingredients. Supplementation with *B. amyloliquefaciens* may further support nutrient utilization and digestive efficiency under alternative feeding systems.

One of the strengths of this study is the integrated evaluation of productive performance, internal and external egg quality, and economic efficiency under a probiotic-supplemented feeding system using locally available alternative feed resources. In addition, the diets were formulated to be iso-nitrogenous and iso-caloric, allowing clearer interpretation of the effects of HI–PCL inclusion levels on laying performance.

However, several limitations should be considered. Water intake and exact probiotic consumption were not measured quantitatively, and carotenoid concentrations in diets and yolks were not analyzed directly. Furthermore, nutrient digestibility, gut microbiota composition, blood biochemical parameters, and long-term production responses were not evaluated. Therefore, the physiological mechanisms underlying improvements in yolk color, nutrient utilization, and production performance could not be fully confirmed.

Future studies should investigate nutrient digestibility, modulation of the intestinal microbiota, immune responses, fatty acid profiles, and carotenoid deposition associated with HI–PCL utilization in laying ducks. Additional studies involving longer feeding periods, different inclusion ratios, and larger commercial-scale production systems are also needed to optimize practical application and evaluate long-term economic sustainability.

Overall, inclusion of the HI–PCL meal blend up to 15% with *B. amyloliquefaciens* supplementation can improve productive performance, enhance yolk color, and increase economic returns in laying Mojosari ducks while maintaining acceptable egg quality. These findings support the potential of locally based alternative feed resources as sustainable, economically beneficial strategies for laying duck production systems.

## DATA AVAILABILITY

The supplementary data can be made available from the corresponding author upon request.

## AUTHORS’ CONTRIBUTIONS

WW: Conceived and designed the study, supervised the research activities, and finalized the manuscript. YH: Contributed to the experimental design and data interpretation. RA: Conducted the animal experiment and collected the data. FM and SYA: Performed laboratory analyses and assisted in data processing. WDR: Carried out the statistical analysis. SR and MR: Contributed to manuscript drafting. All authors have read and approved the final version of the manuscript.
